# Translation and cross-cultural adaptation of the pediatric sleep questionnaire (PSQ*) into Brazilian Portuguese

**DOI:** 10.1016/j.bjorl.2021.03.009

**Published:** 2021-04-20

**Authors:** Carlos Alexandre Necchi Martins, Mayara Moreira de Deus, Isabela Conti Abile, Denny Marcos Garcia, Wilma Terezinha Anselmo-Lima, Carolina Sponchiado Miura, Fabiana Cardoso Pereira Valera

**Affiliations:** Universidade de São Paulo, Faculdade de Medicina de Ribeirão Preto, Divisão de Otorrinolaringologia, Departamento de Oftalmologia, Otorrinolaringologia e Cirurgia de Cabeça e Pescoço, Ribeirão Preto, SP, Brazil

**Keywords:** PSQ, Apnea, Children, Obstructive sleep apnea, Questionnaire

## Abstract

**Introduction:**

Obstructive sleep apnea is a frequent entity in childhood that can lead to important consequences for the health and children's quality of live. Polysomnography is the gold-standard exam to diagnose obstructive sleep apnea, but it is expensive, complex, and poorly affordable in Brazil. The pediatric sleep questionnaire has shown to be a valuable screening test for obstructive sleep apnea. It is a simple questionnaire with good sensitivity and specificity compared to polysomnography in the countries where it has been validated.

**Objective:**

Translation and cross-cultural adaptation of the pediatric sleep questionnaire (PSQ), into Brazilian Portuguese.

**Methods:**

The translation of the PSQ into Brazilian Portuguese was carried out in accordance with good practices. The validated and adapted questionnaire was applied to parents/caregivers of 60 children (40 of them with obstructive sleep apnea and 20 controls) aged 2–18 years. Retest was applied to 30 children with obstructive sleep apnea. The following tests were performed: internal consistency, test–retest, validation of questionnaire (the latter by ROC curve).

**Results:**

Brazilian PSQ has shown high internal consistency by Cronbach's alpha (0.86 for the total test, 0.83 for subscale “snoring”, 0.64 for “sleepiness” and 0.65 for “behavior”). Test–retest presented a correlation of 0.89 for subscale “snoring”, 0.93 for “sleepiness” and 0.86 for “behavior”. Accuracy by ROC curve was 0.99. Nine was considered the optimal value to discriminate patients with obstructive sleep apnea from controls, with a sensitivity of 0.92 and specificity of 1.0.

**Conclusion:**

The translation and cross-cultural adaptation of the PSQ into Brazilian Portuguese proved to be successful. In places with difficult access to polysomnography, PSQ can be a useful tool in screening and follow-up of children with obstructive sleep apnea.

## Highlights

• The Pediatric Sleep Questionnaire (PSQ) is a good screening test for OSA, with good sensitivity and specificity when compared to polysomnography in the countries where it has been validated.
• The translation and cross-cultural adaptation of the PSQ into Brazilian Portuguese showed high internal consistency, high agreement in the test-retest and high accuracy.
• The value of 9.0 was considered ideal to differentiate between patients with OSA and controls, with a sensitivity of 0.92 and specificity of 1.0.
• In places with difficult access to polysomnography, the PSQ can be a useful tool in the diagnostic suspicion and follow-up of children with OSA.

## Introduction

Obstructive sleep apnea (OSA) is a relatively common disease in children, with prevalence between 1% and 5%.^1^ It is associated with considerable morbidity, which results in high medical expenses and treatments,^2^ and negative consequences for the health and quality of life of patients and their families.^3^, ^4^ It is known that OSA in children can lead to cardiovascular (predisposition to systemic arterial hypertension),^5^ somatic (decreased somatic growth),^6^ and neurocognitive impairment (inattention at school, hyperactivity and aggressiveness).^7^, ^8^

Currently, the gold standard test for pediatric OSA is polysomnography^1^ performed in a sleep laboratory, assisted by a polysomnography technician, and with at least 06 h of monitoring. The following parameters are evaluated during sleep: electrooculography, electroencephalography, nasal and oral flow cannulas and thermistors, electrocardiography, pulse oximeter, and muscle electrodes. However, it is a time-consuming and expensive test that requires specialized equipment and personnel and often has long waiting lines.^9^ It is poorly accessible to the general population, making it difficult to diagnose, monitor, and carry out quality care and research on this subject.

The most common treatment for OSA children is adenotonsillectomy.^1^ However, some studies have shown increasing rates of residual OSA after surgery. Brietzke and Gallagner^10^ published a meta-analysis in 2006 in which the success rate of adenotonsillectomy was 82.9%, with 17.1% of patients requiring new treatment. In 2009, Friedman et al.^11^ published a systematic review showing a 59.8% success rate for adenotonsillectomy and, more recently, Lee et al.^12^ published in 2016 a systematic review of 51 studies, with 3413 subjects showing a 51% success rate for adenotonsillectomy, a rate that decreases to 34% among obese children.

All these facts together reinforce the importance of the correct diagnosis of OSA in children, but on the other hand, these factors show the difficulty of accessing polysomnography in some health systems, such as the Brazilian one. In this sense, the questionnaire tests have emerged with some screening functionality for the diagnosis of OSA, as they are easily applied, fast and cost-effective.

There are several questionnaires already published in the literature for this purpose. In a recent comparative study, Burghard et al.^13^ identified 4 that were considered practical and useful as screening for OSA in children. The authors also reinforced that, among the 4 tests, the pediatric sleep questionnaire (PSQ) and the sleep clinical record (SCR) also had the advantage of following the recommendations of the European Respiratory Society Task Force, 2016.^14^

The PSQ version of sleep-related breathing disorder scale (SBD-PSQ) is a questionnaire validated in English in 2000 by Chervin et al.,^15^ in order to facilitate clinical research related to sleep respiratory disorders in children and also access to diagnosis. This questionnaire, in its native language, showed sensitivity between 81% and 85% and sensitivity of 87% in identifying children with moderate to severe OSA compared to polysomnography. The PSQ has already been translated into 12 languages,^16^, ^17^ including Portuguese from Portugal.^18^ Unfortunately, the Portuguese version has linguistic variations that hinder its use in Brazil, particularly in poorer populations.

The aim of this work was the translation and cross-cultural adaptation of the PSQ into Brazilian Portuguese. This version will serve as a screening for the diagnosis of children with OSA, as well as to assess clinical improvement after treatment, which will be extremely important, especially in care centers without access to polysomnography in Brazil, in addition to facilitating research related to childhood sleep respiratory disorders at this institution and others in Brazil.

## Methods

### The original questionnaires

The PSQ version of sleep-related respiratory disorders (SRBD-PSQ) validated by Chervin et al.^15^ has 22 questions aimed at children aged 2–18 years with suspected sleep-disordered breathing. The questionnaire can be self-reported by the child's guardian and is divided into 3 domains: snoring with 9 items, sleepiness with 7 items, and behavior with 6 items. The questions assess the presence or absence of common symptoms, such as snoring, witnessed sleep apneas, difficulty breathing during sleep, daytime sleepiness, inattention and hyperactivity. Positive responses have a score of 1 and negative responses (“no” or “don’t know”) have a score of 0. According to the American authors, a value of 8 or more positive responses is suggestive of childhood sleep apnea.

### PSQ translation

The translation of the questionnaire into Brazilian Portuguese was carried out according to the methodology proposed by Sagheri et al.,^19^ consisting of the following steps:

(1) Independent translation of the questionnaire performed by two bilingual native speakers of Brazilian Portuguese. One of the translators must be familiar with the field of research and be from the medical field (specialist) and the other must not be.
(2) Establishment of a consensual version between the two translated versions. For such, the two translators discuss the best final version, and this discussion must be recorded by an observer.
(3) Reverse translation into English by 2 non-specialist translators native speakers of English, also independently, and who are not familiar with the original English version.
(4) Evaluation of the version by a committee of experts, consisting of health professionals, and all the translators involved, for the development of a pre-final version for field tests. The aim is to ensure accurate translation and appropriate cultural adaptation.

During the committee meeting, small discrepancies between the different versions were assessed, confirming that the Portuguese and English translations were adequate. The committee then decided on the final version for testing on patients. The final version considered the conceptual translation with the simplest possible language, so that it could be applied to the general population, at different levels of education.

This study was approved by the Research Ethics Committee of the Hospital das Clínicas, Faculdade de Medicina de Ribeirão Preto.

### Cross-cultural validation of the PSQ

In order to assess the clarity, adequacy and cultural relevance of the Brazilian Portuguese version, the translated version was applied to the parents/guardians of a sample of 60 children aged 2–18 years, with 40 children followed up at the Specialized Center for Mouth Breathing at Hospital das Clínicas de Ribeirão Preto, diagnosed with OSA confirmed by polysomnography, and 20 children followed up at other outpatient clinics at the same hospital, whose parents spontaneously denied that their kids had breathing problems during sleep. Children with severe physical and mental impairment or parents/guardians who did not know how to read/write were excluded from the study.

In a group of 30 of these children, the questionnaire was completed again after an average period of 15 days to evaluate the test–retest.

### Statistical analysis

For the population studied, only the descriptive data analysis was performed, and no comparative statistical analysis was applied between the groups.

Regarding the completed questionnaires, the following statistical tests were applied to assess their performance:

- Internal consistency, evaluating the homogeneity between the different items of the questionnaire with the total questionnaire using the Cronbach's alpha test. Values between 0.5 and 0.7 represent moderate reliability, and equal to or greater than 0.7 represent high reliability.^20^
- Test–retest of patients who completed the questionnaire twice, using the Kappa concordance test, where values between 0.4 and 0.6 show moderate agreement; between 0.6 and 0.8 high agreement, and above 0.8 almost perfect agreement. For the analysis of subdomains, the intraclass correlation coefficient (ICC) was used, adopting the same values above for the analysis.
- Content validation, measured using Pearson's correlation test for each item of the questionnaire, compared to the total score value. Values above 0.3 have a moderate correlation and above 0.8 a strong correlation.^21^
- Questionnaire validation, measuring the accuracy of the questionnaire to differentiate OSA children from control children. For such, a ROC curve test was performed, and the area under the curve (AUC) was determined: the closer to 1.0, the greater the questionnaire accuracy to differentiate the groups. With this analysis, the best cut-off value for the questionnaire was also obtained, as well as the sensitivity, specificity, and accuracy for this value to differentiate the OSA group from the control group.

In all analyzes, the R program (version 3.6.3) was used. The value was considered statistically significant when *p* < 0.05.

## Results

### Translation/reverse translation

The translation and reverse translation process took place with the participation of two people in each phase, respectively. After the execution of these phases, a remote meeting was held between the members and the research coordinator (total of 6 people), which is recorded on video. After consensus among all members, the final version (Table 1) was presented to the guardians of the evaluated children.

**Table 1:** Final version of the PSQ translated into Brazilian Portuguese.

PSQ – versão português Brasil	Sim	Não	Não sei
*Durante o sono, seu filho:*			
A1. ronca mais que a metade do tempo	□	□	□
A2. sempre ronca	□	□	□
A3. ronca alto	□	□	□
A4. tem a respiração profunda ou ruidosa	□	□	□
A5. Tem dificuldade em respirar ou se *esforça para respirar?*	□	□	□
A6. Você alguma vez já viu seu filho (ou filha) parar de respirar durante o sono?	□	□	□
*O seu filho (ou filha):*			
A7. tende a respirar com a boca aberta durante o dia	□	□	□
A8. acorda com a boca seca	□	□	□
A9. Faz xixi na cama de vez em quando	□	□	□

*O seu filho (ou filha):*			
B1. Acorda cansado de manhã?	□	□	□
B2. em problema de sonolência durante o dia?	□	□	□
B3. Algum(a) professor(a) ou outra pessoa já comentou que seu filho parece sonolento durante o dia?	□	□	□
B4. É difícil acordar seu filho de manhã?	□	□	□
B5. Seu filho acorda com dor de cabeça de manhã?	□	□	□
B6. Seu filho parou de crescer normalmente em algum momento desde o nascimento?	□	□	□
B7. Seu filho está acima do peso?	□	□	□

*Seu filho com frequência:*			
C1. Parece não ouvir quando falam diretamente com ele	□	□	□
C2. Tem dificuldade em organizar tarefas e atividades	□	□	□
C3. É facilmente distraído por estímulos alheios	□	□	□
C4. Fica com as mãos ou pés inquietos ou fica agitado quando sentado	□	□	□
C5. Não para quieto ou frequentemente age como se estivesse ligado na tomada	□	□	□
C6. Interrompe as pessoas ou se intromete em conversas ou brincadeiras	□	□	□

### Studied population

The sample consisted of 60 children, including 39 males (65%) and 21 females (35%), aged between 2 and 17 years (mean 7.9 ± 4.0 years). Of these, 40 children had OSA confirmed by polysomnography and 20 had no spontaneous respiratory complaints. One of the parents/guardians of the participants (usually the mother) agreed to answer the questionnaire and gave written informed consent prior to its application. In 30 children diagnosed with OSA, the same guardian retested the questionnaire, with an average interval of 15 days between the interviews.

In all 60 applications of the test, the guardian did not report any difficulty in answering any question in the questionnaire. In all cases, the parent/guardian took less than 10 min to complete the test.

### Internal consistency

The reliability analysis (or internal consistency) of the study was performed using the Cronbach's alpha coefficient. Analyzes were performed for each subdomain and for the entire questionnaire. The entire questionnaire (PSQ – Brazil) was highly reliable, with Cronbach's alpha of 0.86 (95% CI: 0.82–0.91). The values are shown in Table 2.

**Table 2:** Internal consistency of the PSQ translated into Brazilian Portuguese, assessed using Cronbach's alpha.

Variables	Number of items	Cronbach’s alpha (95% CI)
Subdomain Snorring	9	0.83 (0.76–0.89)
Subdomain Spleepiness	7	0.64 (0.51–0.78)
Subdomain Behaviour	6	0.65 (0.51–0.79)

Total	22	0.86 (0.82–0.91)

Regarding the subdomains, “Snoring” was highly reliable, with Cronbach's alpha of 0.83 (95% CI: 0.76–0.89). The subdomains “Sleepiness” and “Behavior” had moderate reliability, with values of 0.64 (95% CI: 0.51–0.78) and 0.65 (95% CI: 0.51–0.79), respectively.

### Test–retest

Test–retest was assessed using the Kappa Agreement test, and was applied to 30 children, whose parents/guardians answered the same questionnaire on two different days, with an average interval of 15 days between them.

The values corresponding to each question are described in Table 3. Questions A2, B7, and C6 had almost perfect agreement. Questions A5, A8, B5, and C2 had moderate agreement; all other questions had high agreement.

**Table 3:** Reliability assessment of the PSQ test–retest translated into Brazilian Portuguese.

Questão	Kappa	95% CI	*p*-Valor
A1	0.61	0.23–0.99	0.0004
A2	0.92	0.77–1.00	<0.0001
A3	0.71	0.35–1.00	0.0001
A4	0.61	0.20–1.00	0.0010
A5	0.49	0.19–0.80	0.0043
A6	0.65	0.38–0.93	0.0004
A7	0.66	0.31–1.00	0.0003
A8	0.44	0.03–0.85	0.0171
A9	0.78	0.54–1.00	<0.0001

B1	0.69	0.41–0.97	0.0003
B2	0.66	0.39–0.92	0.0003
B3	0.63	0.30–0.95	0.0006
B4	0.78	0.54–1.00	<0.0001
B5	0.56	0.26–0.87	0.0022
B6	0.65	0.02–1.00	0.0002
B7	0.93	0.79–1.00	<0.0001

C1	0.65	0.37–0.93	0.0005
C2	0.59	0.28–0.90	0.0011
C3	0.71	0.33–1.00	0.0001
C4	0.62	0.29–0.96	0.0008
C5	0.66	0.31–1.00	0.0003
C6	0.85	0.65–1.00	<0.0001

The subdomains had high, almost perfect reliability (Table 4). According to the analyzes, the subdomain Snoring had intraclass coefficiency (ICC) value of 0.89 (95% CI: 0.77–0.95); for Drowsiness, the ICC value was 0.93 (95% CI: 0.85–0.97); and for Behavior, the ICC value was 0.86 (95% CI: 0.70–0.94).

**Table 4:** Reliability assessment of the test–retest of the PSQ subdomains translated into Brazilian Portuguese.

Question	ICC	95% CI	*p*-value
Subdomain Snorring	0.89	0.77–0.95	<0.0001
Subdomain Spleepiness	0.93	0.85–0.97	<0.0001
Subdomain Behaviour	0.86	0.70–0.94	<0.0001

ICC, intraclass correlation coefficient.

### Content validation

For content validation, Pearson's correlation test was used for each item of the questionnaire, compared with total score. All questions showed moderate (>0.3 and <0.8) and strong (≥0.8) agreement, except for questions B6 (interruption of somatic speed of growth) and A9 (nocturnal enuresis), which had no significant correlation. None of the items showed a strong correlation (Table 5).

**Table 5:** Pearson's correlation coefficient (*r*) for each question, as well as 95% CI and corresponding *p*-value.

Question	*r*-Pearson	95% CI	*p*-Value
A1.	0.75	0.62–0.85	<0.0001
A2.	0.63	0.44–0.76	<0.0001
A3.	0.57	0.37–0.72	<0.0001
A4.	0.79	0.66–0.87	<0.0001
A5.	0.71	0.56–0.82	<0.0001
A6.	0.58	0.38–0.73	<0.0001
A7.	0.44	0.21–0.63	0.0004
A8.	0.41	0.18–0.61	0.0011
A9.	0.25	−0.01–0.47	0.0588^a^

B1.	0.51	0.30–0.68	<0.0001
B2.	0.54	0.33–0.70	<0.0001
B3.	0.51	0.29–0.67	<0.0001
B4.	0.34	0.10–0.55	0.0072
B5.	0.52	0.31–0.69	<0.0001
B6.	0.12	−0.13–0.37	0.3441^a^
B7.	0.33	0.08–0.55	0.0102

C1.	0.52	0.31–0.69	<0.0001
C2.	0.65	0.48–0.78	<0.0001
C3.	0.57	0.37–0.72	<0.0001
C4.	0.48	0.25–0.65	0.0001
C5.	0.42	0.19–0.61	0.0008
C6.	0.39	0.15–0.59	0.0020

a Not significant.

### Questionnaire validation – ROC curve

**Figure 1:**
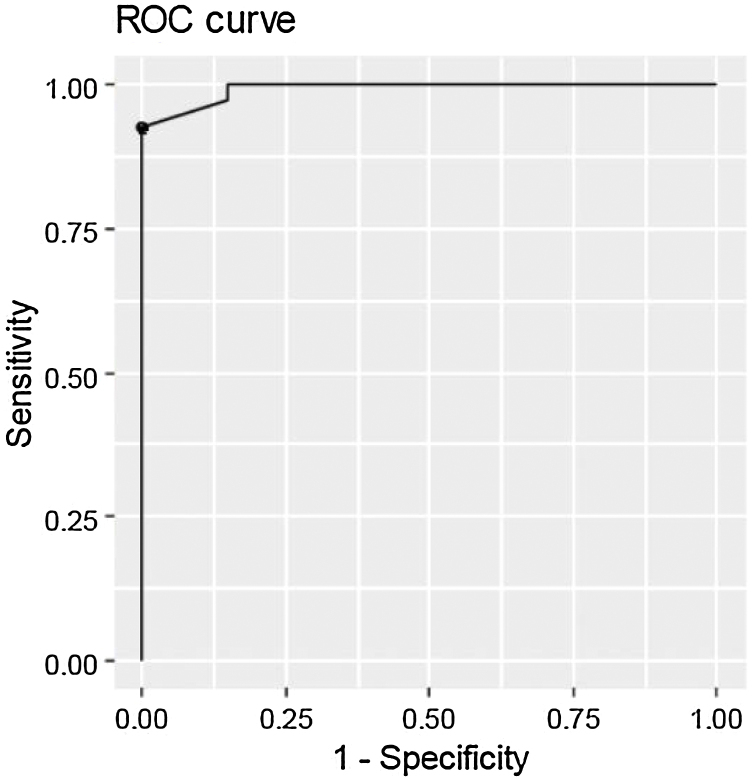
Figure representing the accuracy of the PSQ translated into Brazilian Portuguese in differentiating children with OSA and controls. The ROC curve was almost perfect, with the area under the curve (AUC) = 0.99, and identified the optimum cut-off point as 9 points. At this point, the sensitivity was 0.92, specificity was 1.00, and accuracy was 0.95 to differentiate one group from another.

Finally, we performed the ROC curve test to assess the accuracy of the questionnaire in differentiating between AOS children and control children in our cohort. The questionnaire result was an AUC = 0.99; that is, almost perfect (Fig. 1). The same analysis also identified the best cut-off score, which was considered 9.0. This value had a sensitivity of 0.92; specificity of 1.0, and accuracy of 0.95, that is, children with a score equal to or above 9.0 have a high probability of having OSA, while those who obtain values below 9.0 have a low probability of having sleep respiratory disorder.

The subdomains also had good accuracy in differentiating one group from the other: “Snoring” AUC = 0.94; “Drowsiness” AUC = 0.85; and “Behavior” AUC = 0.92.

## Discussion

Early diagnosis, treatment, and adequate follow-up of OSA are essential to avoid the morbidity associated with this disease. However, in clinical practice, we face difficulty in carrying out the gold standard test (polysomnography) for diagnosis, as it is an expensive, time-consuming and inaccessible test, especially for the pediatric population.

In this context, new tools are needed to assist in screening for OSA in children, and diagnostic questionnaires emerge as simple, inexpensive, and easy-to-apply alternatives.

Among the existing questionnaires are the sleep clinical record (SCR),^22^ which is quite complete, but complex and time-consuming,^13^ the OSA-18, which assesses the impact of obstructive symptoms on quality of life, but as a method diagnosis has low sensitivity and specificity,^23^ and the PSQ. The PSQ stands out for having high sensitivity and specificity and for being easy and quick to apply, without the need for a doctor or trained professional.

Since its validation in 2000, the PSQ has been translated into several languages, including Portuguese spoken in Portugal.^18^ However, the linguistic difference between the two countries makes it difficult to understand some questions, particularly among individuals with a lower socioeconomic status. A clear example of this difference is present in domain A (Snoring). In the Portuguese version, the word “ressonar” is present in 3 questions, while in Portuguese spoken in Brazil the verb “roncar” would be the equivalent word most frequently used. This difficulty in the self-applied use of this questionnaire among patients in our service motivated us to carry out this study, which aimed at the translation and cross-cultural adaptation specific to the Portuguese spoken in Brazil.

For the translation of the original PSQ validated by Chervin et al.,^15^ we strictly follow the methodology proposed by Sagheri et al.^19^ Then, it was applied to the parents or guardians of 60 children aged between 2 and 18 years, among whom 40 had OSA confirmed in polysomnography and 20 were asymptomatic. None of the parents/guardians reported difficulties in understanding any question in the version presented by us. Still, the PSQ – Brazil showed high accuracy in differentiating between the tested children those with OSA from the controls, as evidenced by the ROC curve test (AUC = 0.99), with a sensitivity of 0.92 and specificity of 1, when the cut-off value of 9 is used. This demonstrates that children with PSQ values up to 9 have a low probability of having OSA.

Also noteworthy is the high reliability of the Brazilian version of the questionnaire, evidenced by the Cronbach's alpha value of 0.86, corroborating the findings of the original version and other versions, such as the French and Portuguese versions.^15^, ^16^, ^18^

Regarding the test stability, the subdomains showed almost perfect reliability, with ICC values of 0.89 for the Snoring subdomain; 0.93 for Drowsiness; and 0.86 for Behavior.

The study has some limitations, such as its application to children followed at a specialized tertiary center and, therefore, may present more severe and exuberant forms of the disease. Still, all children included in the study are users of the Brazilian Unified Health System (SUS), and may not adequately represent all socioeconomic niches of the Brazilian population. Finally, Brazil is a continental country with linguistic differences between each region, and the result observed in our center may not be the same in other Brazilian regions.

Still, the translation study and the cross-cultural adaptation of the PSQ for Brazil showed values similar to those seen in other translations in the world, as well as the original American version, and therefore can be used as a reliable screening test in Brazilian children with suspected OSA.

## Conclusion

The translation and cross-cultural adaptation of the PSQ into Brazilian Portuguese proved to be successful with evidence of strong correlation with the results obtained in the original English version. In places with difficult access to polysomnography, PSQ can be a useful tool in the diagnostic suspicion and follow-up of children with OSA.

## Conflicts of interest

The authors declare no conflicts of interest.
